# Dental Optical Coherence Tomography

**DOI:** 10.3390/s130708928

**Published:** 2013-07-12

**Authors:** Yao-Sheng Hsieh, Yi-Ching Ho, Shyh-Yuan Lee, Ching-Cheng Chuang, Jui-che Tsai, Kun-Feng Lin, Chia-Wei Sun

**Affiliations:** 1 Graduate Institute of Photonics and Optoelectronics and Department of Electrical Engineering, National Taiwan University, Taipei 106, Taiwan; E-Mails: d01941022@ntu.edu.tw (Y.-S.H.); jctsai@cc.ee.ntu.edu.tw (J.-C.T.); 2 Department of Photonics and Biomedical Optical Imaging Lab, National Chiao Tung University, Hsinchu 300, Taiwan; E-Mails: d95543004@ntu.edu.tw (C.-C.C.); lkf169@gmail.com (K.-F.L.); 3 School of Dentistry, National Yang-Ming University, Taipei 112, Taiwan; E-Mails: rebecca1112@gmail.com (Y.-C.H.); sylee@ym.edu.tw (S.-Y.L.); 4 Department of Dentistry, National Yang-Ming University Hospital, I-Lan 260, Taiwan; 5 Department of Stomatology, Taipei Veterans General Hospital, Taipei 112, Taiwan; 6 Biophotonics and Molecular Imaging Research Center, National Yang-Ming University, Taipei 112, Taiwan

**Keywords:** optical coherence tomography, dental imaging, oral diagnosis, tooth imaging

## Abstract

This review paper describes the applications of dental optical coherence tomography (OCT) in oral tissue images, caries, periodontal disease and oral cancer. The background of OCT, including basic theory, system setup, light sources, spatial resolution and system limitations, is provided. The comparisons between OCT and other clinical oral diagnostic methods are also discussed.

## 1. Introduction

Optical coherence tomography (OCT) was first reported by Fujimoto *et al.* in 1991 [1]. OCT has been widely used in numerous clinical applications, including gastroenterology [2–4], ophthalmology [5–7], dermatology [8,9], and dentistry [10,11]. OCT is a non-invasive, non-radiative optical diagnostic tool based on interferometers. By using a low-coherence broadband near-infrared light source, it is possible to obtain excellent spatial resolution (∼20 μm) and real-time images [12,13]. OCT was first applied *in vitro* in human retina and in atherosclerotic plaque [1,14]. It is an optical imaging technique that enables cross-sectional imaging of microstructures of tissue *in situ.* OCT can provide “optical biopsy” without the need for excision and processing of specimens as in conventional biopsy and histopathology. With improvement of optical specifications and system capabilities, OCT demonstrates great potentials in research topics and clinical applications to date.

Over the past decade, many functional OCT systems, such as Doppler OCT (DOCT) [15,16], polarization sensitive OCT (PS-OCT) [17–19], endoscopic OCT [20,21] and acoustic OCT [22,23], were reported for new biomedical research applications These functional systems provide not only structure images but also the specific optical characteristics, including blood flow velocity and tissue orientation. Moreover, deeper transmission depth is achieved with combination of fluorescence [24,25]. Indeed, these optional functions promote the efficiency of diagnosis of OCT.

Application of OCT in dentistry has become very popular. The first *in vitro* images of dental hard and soft tissues in a porcine model were reported in 1998 [26]. Later, the *in vivo* imaging of human dental tissue was presented [27]. The oral cavity consists of three main parts: (1) hard tissue, including tooth and alveolar bone, (2) soft tissue, including mucosa and gingiva tissues, and (3) periodontal tissues [28]. The traditional diagnosis of caries is based on examination using dental exploration and radiographs. The diagnosis of periodontal disease needs the examination of periodontal probes. The poor sensitivity and reliability of periodontal probing make it difficult for dentists to monitor the progression of periodontal destruction and the treatment outcome [29]. Radiography may be the most popular diagnostic tool recently. However, radiography provides only two-dimensional images. The caries or bone structure on the buccal and lingual sides of teeth may be superimposed with tooth structures or normal anatomic structures. The radiation exposure of radiographic techniques is also a great concern. Furthermore, early detection of caries, periodontal disease and oral cancer is quite difficult with clinical examination or radiographs.

OCT may provide a solution to these problems. Dental OCT detects qualitative and quantitative morphological changes of dental hard and soft tissues *in vivo.* Furthermore, OCT can also be used for early diagnosis of dental diseases, including caries, periodontal disease and oral cancer, because of the excellent spatial resolution. Early detection and treatment can increase the survival rates of teeth and patients. Three-dimensional imaging ability is another advantage of dental OCT. It helps clinicians to locate problems in soft and hard tissues more accurately and rapidly.

This review paper discusses the development of dental OCT. The applications of OCT in oral tissue images, tooth decay, periodontal disease and oral cancer are also reviewed. A systematic comparison between dental OCT and other diagnostic methods, including periodontal probing, radiography, fluorescence and Raman spectroscopy, is also presented.

## 2. Systems

### 2.1. OCT System and Important Specifications

**Figure 1.**
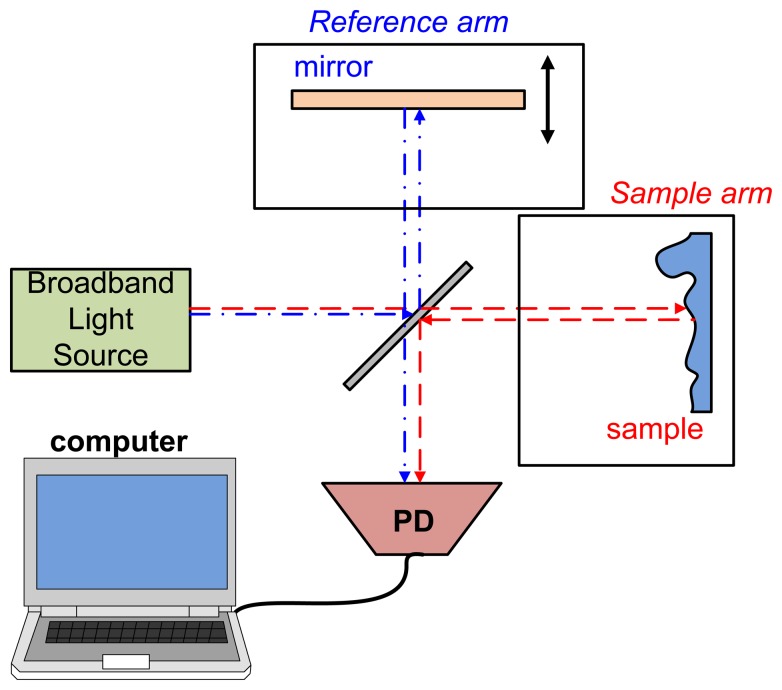
System setup of TD-OCT (PD: photodiode).

OCT is an interferometer-based system with a low coherence length broadband light source. The lights reflect from the sample and reference arms interference within a Michelson or Mach-Zehnder interferometer. This interference signal is acquired by a photodiode (PD) or charge-coupled device (CCD) that is dependent on the type of OCT. Figure 1 shows the first OCT type, time domain OCT (TD-OCT). TD-OCT acquires various optical path lengths (OPLs) by moving a reference reflector [30]. The light interference occurs when the OPL of the reference and of lights reflected by samples are the same. Furthermore, the constructive interference (bright lines) arises when the optical path difference (OPD) between two lights is an integer multiple of the wavelength. Therefore, a low coherence length light source is usually used for observing only one interference envelope from a selected depth. The relationship between OPL and OPD is described as Equation (1):

(1) OPD=ΔOPL=Δ(k×n×d)

where *k* is the wave number, *n* is the refractive index of material and *d* is the propagation length in air.

**Figure 2.**
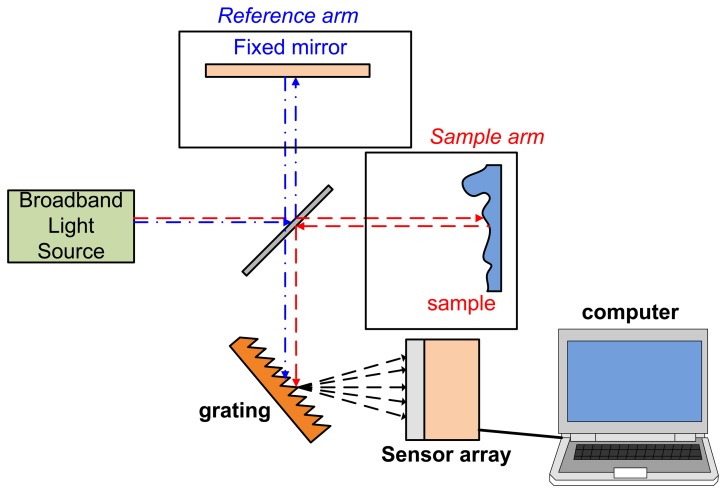
System setup of SD-OCT.

Another common type of OCT is the spectral domain OCT (SD-OCT) or Fourier domain OCT (FD-OCT). Unlike TD-OCT, the OPL in SD-OCT is decided from different wavelengths [30] and no moving reflection mirror is necessary. A SD-OCT system is setup with almost same components as TD-OCT but with an additional grating (for spatial Fourier transform), sensor array (usually CCD array) or spectrometer. A SD-OCT system setup is shown in Figure 2.

The OCT axial resolution is related to the light source coherence length *lc*, which is a function of the light source bandwidth (Δλ). The light source coherence length can be described by Equation (2) [31]:

(2) $$l_{c}=\frac{2c\;\text{ln}\;2}{\pi }\frac{1}{\Delta v}=\frac{2\;\text{ln}\;2}{\pi }\frac{\lambda_{0}^{2}}{\Delta \lambda }\approx 0.44\frac{\lambda_{0}^{2}}{\Delta \lambda }$$

where λ_0_ is the center wavelength. Δλ is also known as the full width at half the maximum (FWHM) of spectrum.

OCT systems with appropriate broadband light sources usually demonstrate excellent axial resolution <20 μm [12,13]. The transverse resolution is decided by the final spot size on the sample. Higher transverse resolution may be achieved with a focused light. The dispersion and phase compensation are also important to an OCT system. Detailed reviews of these advanced technologies can be found in the references [1,7,9,18].

Functional OCT can also provide sample functional information such as blood velocity (by DOCT [16,32]), the organization of tissue structural (by PS-OCT [33,34]). Clinicians may select a suitable type of OCT according to their requirements.

### 2.2. Comparison with Other Dental Diagnostic Methods

Traditionally, the presence of dental disease is assessed using visualization and probing. However, their sensitivity and reliability are questionable. Radiography and dental computed tomography (dental CT) have become the most popular diagnostic methods today. Dental CT could provide three-dimensional images for better diagnosis. Nevertheless, the harmful ionizing radiation of radiography and dental CT limit their usage. Very early diagnosis of disease by radiography is difficult. Recently, several novel techniques have been developed for diagnosis of dental disease, such as a smart ultrasonic devices [29,35–37], LED-based dental optical probes [38], and laser fluorescence [39,40]. Raman and laser fluorescence spectrometers may also be applied in the detection of dental disease but are still under investigation. Raman spectrometers can measure the mineral and chemical content of tissues *in vitro*. The laser fluorescence spectrometer is reported as a tool to detect and quantify caries. Lack of diagnostic consistency would be a problem. The presence of bacteria, electrolytic solutions, and blood considerably influence the intensity of fluorescence [39,40]. Table 1 shows a comparison between dental OCT and other dental diagnostic methods used today. In conclusion, OCT is an effective diagnostic tool because it is a noninvasive, nondestructive, non-radiated, and real-time monitoring method.

## 3. Applications of Dental OCT

Early OCT studies focused mainly on the topics of dental soft and hard tissue morphology because of the limitation of system size and light source manufacture technology [45–47]. Nowadays, with well-developed components, this powerful tool could be applied in last decade in advanced diagnosis problems such as tooth decay and periodontal disease. OCT is not only an “imaging tool” but also an important and non-invasive method for early detection of oral disease. The important clinical applications of dental OCT, including tooth decay, periodontal disease and oral cancer, will be reviewed.

### 3.1. Tissue Images

**Figure 3.**
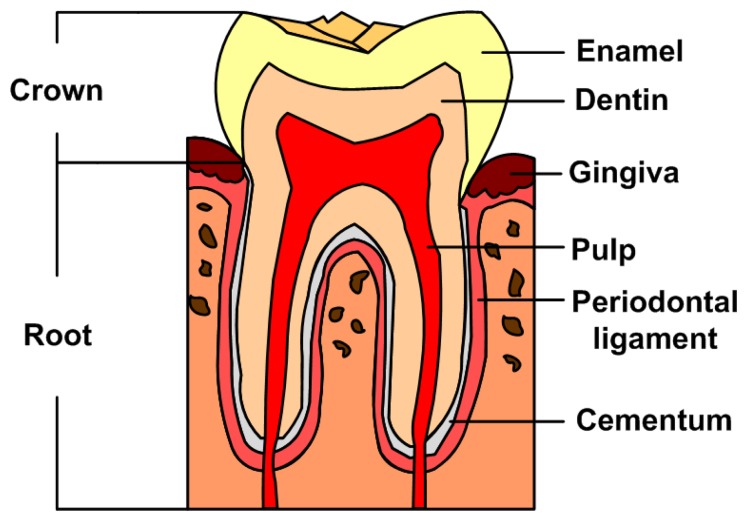
Schematic diagram of a tooth structure.

Figure 3 illustrates a tooth structure. Colson *et al.* first reported the 1,310 nm TD-OCT image of teeth and compared them with a photomicrograph under 17 μm resolution [26]. They only examined the characteristics of the oral structure surface because of the insufficient penetrating depth. The *in vitro* images of enamel-cementum and gingiva-tooth interfaces in a porcine model were shown. Otis *et al.* presented the first *in vivo* OCT images of human dental tissues [47]. The axial resolution was 12 μm with 1,310 nm center wavelength. They obtained a smaller but deeper (3 mm) tooth image. Their OCT images provided visual recording of the dentin-enamel junction (DEJ) and periodontal structures. Feldchteine *et al.* demonstrated that hard palate mucosa and gingiva mucosa could be visualized [45]. The OCT images showed the hard palate mucosa. The squamous epithelium appears as the 170 mm top layer above the 200 mm thick lamina propria. OCT images also displayed gingiva mucosa to a depth of 500 μm, although the epithelium and lamina propria were not well differentiated in their scans. Moreover, they also presented the polarization imaging of normal dental hard tissue. OCT images in normal polarization scan mode showed that enamel, dentin, and DEJ were clearly visible. Warren *et al.* provided more detail tooth structure along the vertical axis [48]. The axial structure from enamel to dentin and cementum to dentin was revealed.

**Figure 4.**
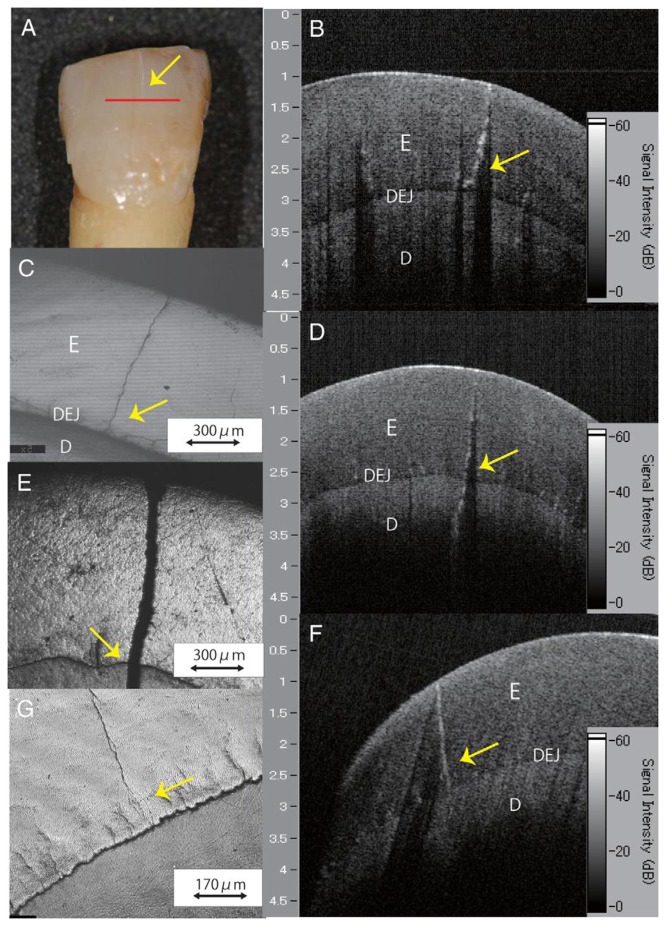
Images of a distinct enamel crack. (**A**) A visual examination of enamel crack. (**B**) A SS-OCT image along the red line in (A). The crack extended to the DEJ. (**C**) A CLSM image corresponding to the cross-sectioned enamel crack along the red line in (A). The crack ended up to the DEJ. (**D**) A SS-OCT image of a sample determined as a deep enamel crack transillumination. The crack was seen extending beyond the DEJ. (**E**) A CLSM image corresponding to the cross-sectioned enamel crack in (D). The crack penetrated deep into the dentin. (**F**) A SS-OCT image of a sample determined as a superficial enamel crack with transillumination. The crack had extended into the DEJ. (**G**) A CLSM image corresponding to the cross-sectioned enamel crack in (F). The crack had not extended into the DEJ (E: enamel; D: dentine; DEJ: dentin-enamel junction) (reprinted from reference [49]).

**Figure 5.**
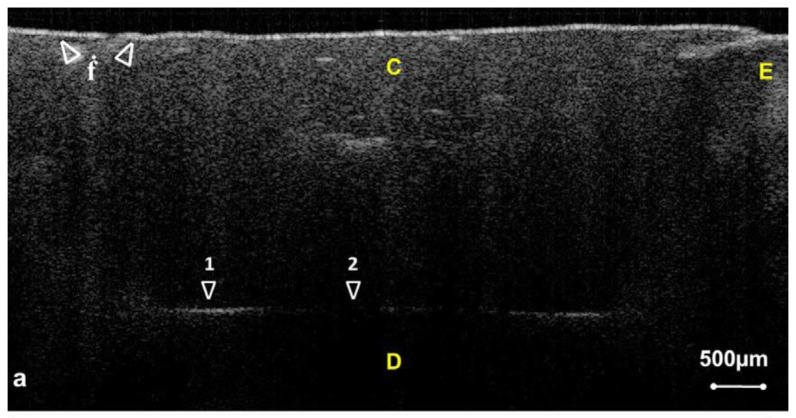
OCT images revealed microleakage between composite resin restoration and the tooth. (C: composite restoration; E: enamel; D: dentin) (reprinted from reference [52]).

**Figure 6.**
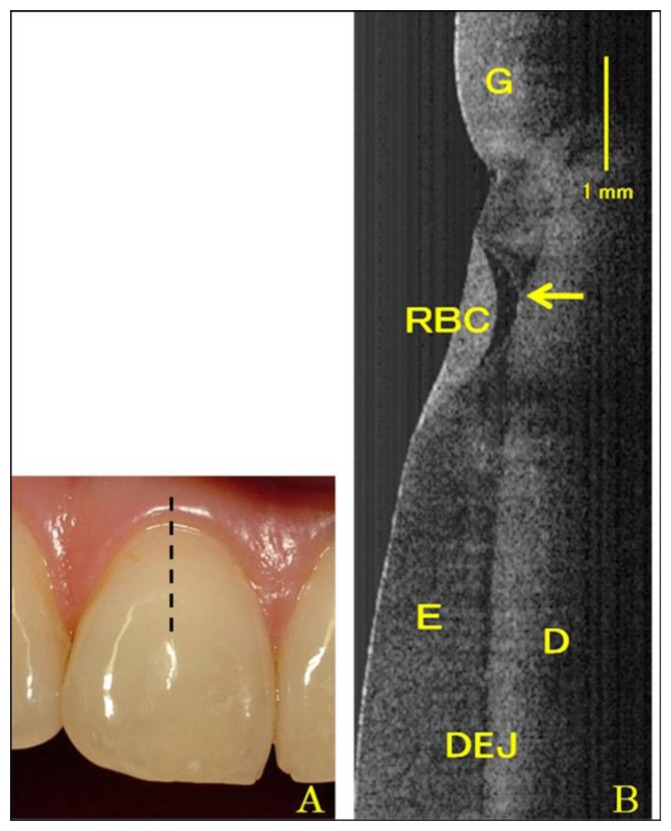
Photogragh (**A**) and SS-OCT image (**B**) of Class V restoration in the central incisor. Arrow shows microleakage formation beneath resin material (G: gingival; RBC: resin based composite; E: enamel; D: dentin; DEJ: dental enamel junction) (reprinted from reference [54]).

**Figure 7.**
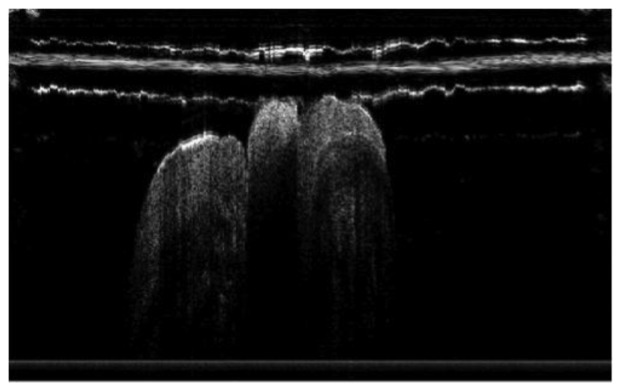
*In vivo* OCT image of microleakage detected by a custom-made dental optical probe (reprinted from reference [55]).

In addition to structure image measurement, OCT is also applied for crack (fracture) [49–51] and microleakage [52–55] detection. The definition of cracks is the “gaps” in the tooth surface, such as enamel cracks. Cracked teeth may lead to extraction if there is no treatment intervention. Imai *et al.* represented the extension of enamel cracks beyond DEJ (Figure 4) [49]. Microleakage means the “gap” between tooth and restorative materials (Figure 5) [52–55]. Ishibashi *et al.* demonstrated the microleakage beneath composite resin restorative material (Figure 6) [54]. Hsieh *et al.* also detected *in vivo* microleakage in OCT images with a custom-made dental optical probe (Figure 7) [55]. They also measured the microleakage at approximately 401 μm × 148 μm in size, which is very close to the real size.

**Figure 8.**
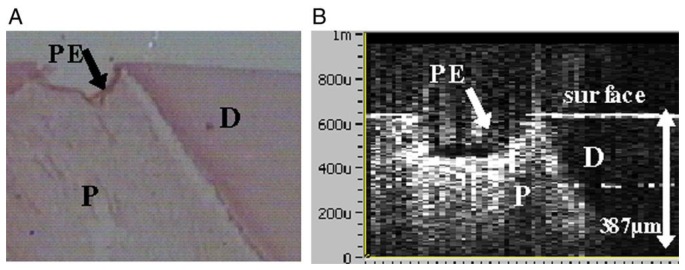
Site of pulp exposure. (**A**) Histologic cross-section of pulp exposure. (**B**) The pulp and dentin were clearly delineated in the OCT image (P: pulp; D: dentin; PE: pulp exposure) (reprinted from reference [56]).

Recently, OCT was also used in imaging of the pulp-dentin complex [56]. The result of this study showed the capacity of OCT to distinguish pulp from dentin (Figure 8). OCT can be used to predict remaining dentin thickness above pulp, and will permit more predictive prognosis of dental treatment.

### 3.2. Tooth Decay: Caries, Attrition and Abrasion

**Figure 9.**
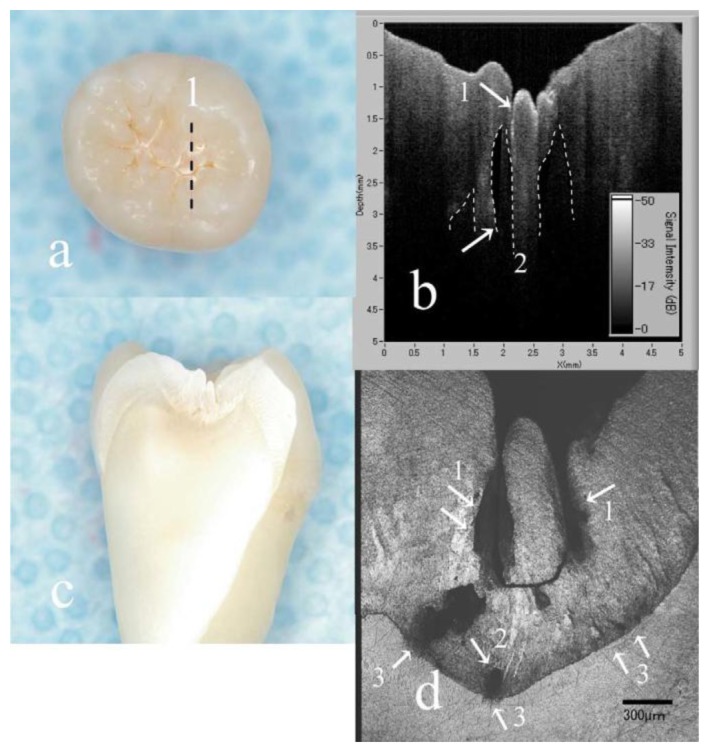
OCT images of the carious lesion and sound regions. (**a**) Visualization of a slightly demineralized tooth. (**b**) SS-OCT image obtained at line 1 in A. The presence of demineralization was determined as a strong scattering signal from the bottom of the fissure (arrow 1). Reflecting OCT signal from the enamel was in some part lacking elliptically beneath the occlusal fissure (arrow 2, dotted line). (**c**) Cross-sectioned view at line 1 in A. (**d**) CLSM image correspond to the cross-sectioned “enamel demineralization” at line 1 in A. CLSM observation clearly confirmed the presence of “hidden lesion” (arrows 1 and 2). Compared with B, the hidden lesion located at the outer enamel was detectable in SS-OCT (arrow 1); however, deeper lesions located near the EDJ could not be visualized in SS-OCT (arrow 2). Since the EDJ vicinity appeared roughened (arrow 3) some of which were reached to the dentine (reprinted from reference [65]).

Caries is an important dental care issue. Caries has high prevalence and wide distribution among ages. The World Health Organization (WHO) revealed that dental caries is still a major public health problem globally and major public health problem in most high-income countries. This disease affected 60%–90% of school-aged children and the vast majority of adults in 2009 [57]. Studies also showed that poor oral hygiene and dental caries may correlate with various systemic diseases, such as systemic infection, kidney inflammation and septicemia [58–61]. The mechanical tooth wear due to attrition and abrasion may also cause the loss of tooth structures. OCT provides the capability for early detection of caries (Figure 9) [62–65]. Because strong birefringence in enamel and anisotropic light propagation through dentinal tubules was observed, many research projects are focused on the application of PS-OCT in caries detection [66–76]. Baumgartner *et al.* presented the first polarization resolved images of dental caries [66–68]. Wang *et al.* measured the birefringence in dentin and enamel and suggested that the enamel rods acted as waveguides [46]. PS-OCT is suitable for the detection of secondary caries, because the scattering properties of restorative materials and dental hard tissue have marked differences [70,71].

**Figure 10.**
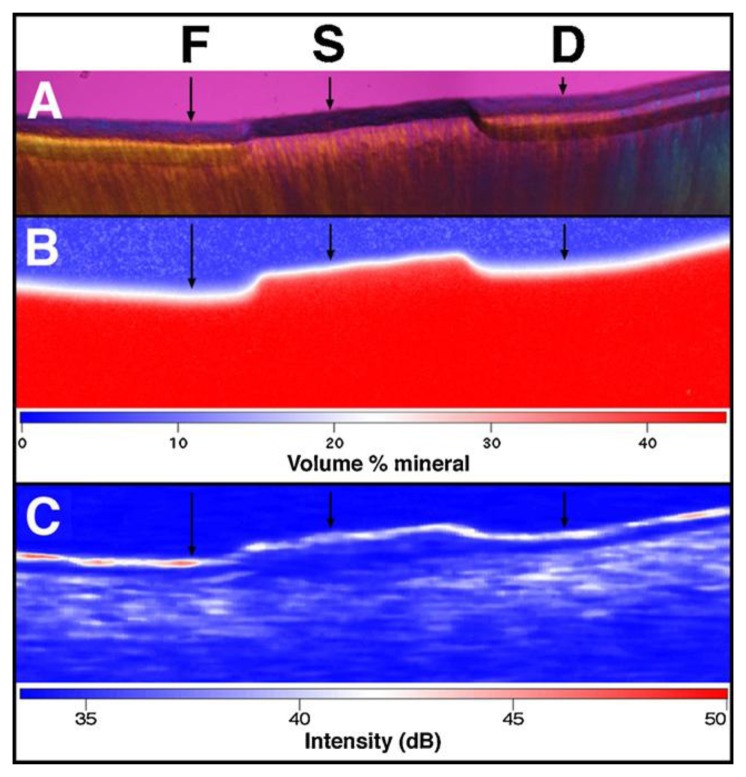
Measuring demineralization by polarized light microscopy (PLM), transverse microradiography (TMR), and PS-OCT images. The black arrows indicated topical fluride (F), protected sound dentin (S), and untreated demineralization area (D). PLM (**A**) shows the lesion depth on both sides (F and D) of the sound protected area. TMR (**B**) and PS-OCT (**C**) images are also shown along with the false color intensity scales. PLM and PS-OCT were superior to TMR as a tool for measuring lesion depth and cementum layer thicknesses because these measurements were much higher sensitivity to mineral loss (reprinted from reference [72]).

Nowadays, PS-OCT is often used for very early caries diagnosis, because it can determine the level of demineralization for early detection of caries (Figure 10) [72–75]. Moreover, recent researches showed that an OCT system with an integrated micromechanical system (MEMS) scanner could obtain a 3D OCT image [76]. This could lead to rapid detection of both early demineralization and more severe lesions.

### 3.3. Periodontal Diseases

**Figure 11.**
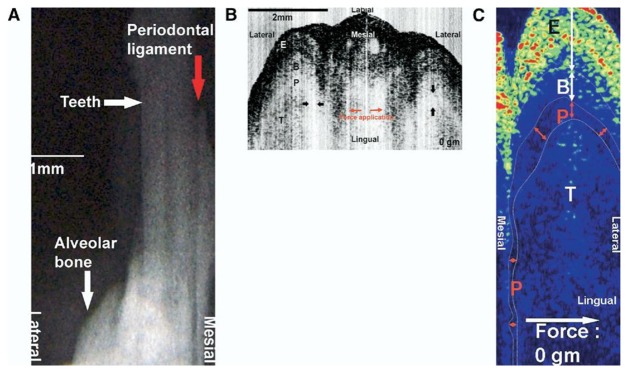
Images of a periodontal ligament. (**A**) Radiograph. (**B**) OCT. (**C**) Logging OCT images; the boundary of each tissue can be identified more clearly (reprinted from reference [82]).

**Figure 12.**
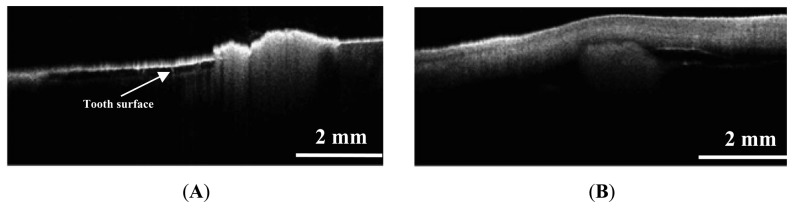
OCT image of subgingival calculus. (**A**) Subgingival calculus without coverage of gingiva. (**B**) Subgingival calculus covered with gigniva (reprinted from reference [28]).

Periodontitis is one of the major chronic infectious diseases in the oral cavity. The prevalence of periodontitis is more than 50% among the population [77,78]. The WHO revealed that tooth loss resulting from severe periodontitis was found in 5%–15% of most worldwide populations in 2003 [79]. Additionally, recent studies have indicated that certain correlations between periodontitis and various systemic diseases exist [80–82]. Colston *et al.* were the first group to apply OCT in the diagnosis of periodontal disease [83,84]. They took *in vitro* images of dental and periodontal tissues from a young porcine model and compared these images to histological sections. Feldchteine *et al.* demonstrated epithelium and lamina propria of gingival mucosa [45]. However, the epithelium and lamina propria were not well differentiated because of the physical limitation. Baek *et al.* represented OCT images of periodontal ligaments during orthodontic movement of rat (Figure 11) [85]. Hsieh *et al.* demonstrated subgingival calculus *in vitro.* Tooth with subgingival calculus covered with 0.8 mm porcine gingiva was measured (Figure 12). Subgingival calculus is one of the pathogenetic factors of periodontal disease, so it is important to remove the residual subgingival calculus [28]. The refractive indices of enamel, dentin, cementum, and calculus were also measured as 1.625 ± 0.024, 1.534 ± 0.029, 1.570 ± 0.021, and 2.097 ± 0.094, respectively. The refractive indices help clinicians to distinguish calculus from normal tissues rapidly and correctly. With the aid of OCT, early detection of periodontal disease and monitoring of periodontal treatment could be very helpful.

### 3.4. Oral Cancer

Oral cancer has become the fourth leading cause of cancer death in males in Taiwan [86]. An estimated 263,900 new cases and 128,000 deaths from oral cavity cancer (including lip cancer) occurred in 2008 worldwide [87]. Oral cancer occurs with an annual incidence of approximately 29,370 cases in the United States [88]. Treatment of oral cancer and the survival rates are directly related to the stage of cancer diagnosed. Early diagnosis permits minimally invasive treatment and greatly improves long-term survival. Wilder-Smith *et al.* reported that OCT could detect neoplasia-related epithelial, sub-epithelial changes throughout carcinogenesis [89]. Jung *et al.* also obtained similar results with better resolution [90]. Moreover, they incorporated three-dimensional images and applied Doppler OCT for better diagnosis. Tsai *et al.* demonstrated swept-source OCT (SS-OCT) had better image qualities than SD-OCT [91,92]. Moreover, by utilizing nanoparticles, OCT could obtain better contrast images at early stage of oral cancer [93,94]. OCT OCT is a good tool for early diagnosis of oral cancer, providing better understanding of pathological mechanisms, predictors of malignant change, risk of tumor recurrence, and predictors of tumor response to therapy [89].

**Figure 13.**
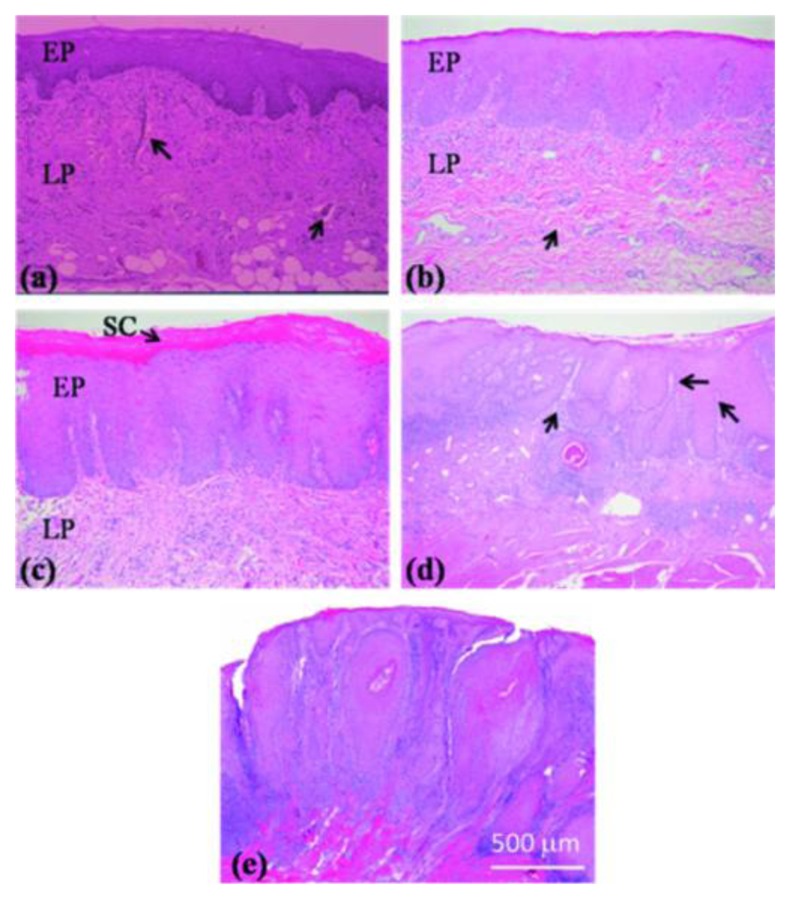
Histological images of the (**a**) normal, (**b**) MiD, (**c**) MoD, (**d**) ES-SCC, and (**e**) WD-SCC samples (reprinted from reference [95]).

**Figure 14.**
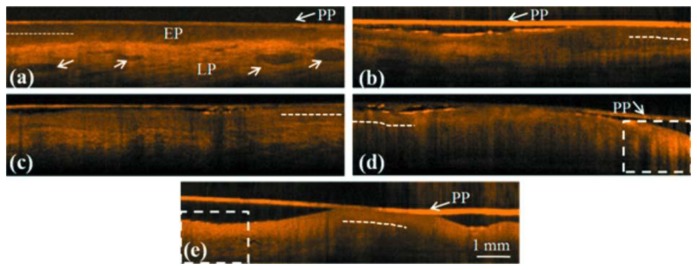
SS-OCT scanned images of the (**a**) normal control and biopsied oral (**b**) MiD, (**c**) MoD, (**d**) ES-SCC, and (**e**) WD-SCC lesions. Their histological images were shown in Figure 13(a–e) (reprinted from reference [95]).

Tsai *et al.* utilized SS-OCT to differentiate different oral carcinogenesis stages, including mild dysplasia (MiD), moderate dysplasia (MoD), early-stage squamous cell carcinoma (ES-SCC), and well-developed SCC (WD-SCC) [95]. Figure 13 shows histological images of normal, MiD, MoD, ES-SCC and WD-SCC. In the normal sample, the epithelium (EP) and lamina propria (LP) layers could be clearly differentiated. Vessels could also be observed in the LP layer. In the MiD stage, the EP layer was thickened, dysplastic cells were found in the lower one-third of EP, and there was an increase in collagen deposition in the LP layer. A thick stratum corneum (SC) layer can be found on EP surface in MoD stage and EP become thicker than in MiD stage. At ES-SCC and WD-SCC stages, the EP/LP boundary disappeared. On the basis of SS-OCT images, oral precancer lesion (MiD and MoD) and oral cancer (ES-SCC and WD-SCC) could be differentiated using OCT (Figure 14). It is beneficial to diagnose oral precancer patient. Minimally invasive treatment could enhance patient's life quality.

A similar analysis was also used for oral submucous fibrosis (OSF). Lee *et al.* measured the thickness of EP and SD for OSF diagnosis [96]. The increasing thickness of EP and DS was thickened by the dysplasia collagen.

### 3.5. Other Dental-Related Diseases

As OCT can delineate changes in epithelium, this imaging tool has potential broad applications in mucosal lesions, including lichen planus, pemphigoid, or vascular lesions. Further research was necessary to provide more detailed images. Studies discussed the OCT image quality with a 1,550 nm light source [97,98]. They revealed the possibility of this application for bone-related disease imaging. Future research should focus on the suitable wavelength of light source of OCT for better observation of oral-related disease.

## 4. Discussion

There are still some limitations on the popularization of dental OCT applications. The first issue is the insufficient scanning range of OCT. Because the scanning range is usually several millimeters, hundreds or thousands of pictures may be necessary for a whole lesion. A well-developed dental optical probe incorporating OCT may overcome this problem. The convenience of dental optical probes enables clinicians to screen the whole lesion and to focus on the specific area rapidly. Second, the low penetration depth also restricts utility for clinical use. The issue of penetration depth is a physics problem. Choosing a high-quality light source may be a solution, however, a high-quality light source will increase the cost of an OCT system.

Besides, there are some factors affecting the performance of OCT in dental applications. Wavelength choice may be the most important factor in these experiments. Within the near-infrared window in biological tissue, the center wavelength determines the maximum depth of penetration in tissue due to the scattering and absorption properties [99]. When the wavelength is under 1,000 nm, the scattering property is the main effective factor because of the similar size of light and particles in tissue. This phenomenon is often analysed by Mie scattering theory. The absorption effect increases after 1,000 nm and reaches the maximum around 1,400 nm. Water in tissue will decay the input of light energy strongly. Therefore, different wavelengths are employed for a variety of samples. For example, an OCT system with 1,550 nm center wavelength is good for hard tissue measurement but not suitable for soft tissue imaging, because the input light will be absorbed by blood or water. In dental application, this system is more appropriate for hard tissue, such as enamel, dentin and alveolar bone, but not for mucosa or gingiva imaging.

The composition and uniformity of sample is another factor that affects experiment results. Samples with rough surfaces or inhomogeneous composition show lower penetration depth and image contrast because of scattering effects. Another important factor is the index difference between sample and background. Optical scattering occurs due to mismatches in refractive index of the different tissue components. Therefore, materials with similar refractive index will demonstrate similar OCT images. For example, the retina layers are difficult to distinguish because of their similar compositions. Functional OCT, including DOCT and PS-OCT, gathers more information in biological tissues. DOCT can provide the blood velocity and inflamed tissue volume information. On the other hand, PS-OCT can be used for structure orientation due to the polarization property.

For *in vivo* measurement, some important issues, including dental optical probe design, data acquisition time and scanning range, should be taken into consideration. A well-design dental optical probe is indispensable for clinical oral imaging. Also, dental optical probes enable three-dimensional imaging.

Data acquisition time is another key factor. OCT can obtain images in seconds. However, low image quality will be observed with faster imaging speeds due to insufficient processing time. Users should select a balance between image quality and acquisition time.

## 5. Conclusions

OCT has become a very important research tool in medical and clinical diagnosis. In dentistry, dental OCT provides the benefits of low cost, non-invasive, non-radiative, and high resolution. Dental OCT demonstrates broad applications in soft and hard tissue imaging and early detection of caries, periodontal disease and oral cancer.

For tissue imaging, OCT can be used for gingiva, periodontal and mucosa imaging. Clinicians can obtain not only the structural images by conventional OCT, but also blood information and structure orientation by functional OCT methods such as DOCT and PS-OCT. With a longer center wavelength, OCT may also apply in bone-related disease imaging.

OCT and PS-OCT represent powerful ability for early diagnosis of caries. Mineral changes at early demineralization stages can be distinguished by PS-OCT. OCT can diagnose periodontal disease and precancerous lesions. Subgingival calculus can also be detected by OCT. Minimum invasive therapy could be performed. Better treatment outcomes and survival rates could be obtained through early detection of oral cancer.

OCT provides images of dental tissue *in situ* and real-time. OCT imaging allows early detection of many oral diseases, including caries, periodontal disease, and oral cancer. In the future, an OCT system with handheld optical probe and minimized system setup should demonstrate its feasibility for telemedicine with a Picture Archiving and Communication System (PACS). This will be helpful in the home nursing care plan in our aging society.

## Figures and Tables

**Table 1.** Comparison between dental OCT and other methods.

**Methods**	**Advantages**	**Disadvantages**
Radiography [29]	Low cost Broad measurement range	Radiative Poor spatial resolution Only 2-D image
Dental-CT [29]	Broad measurement range 3-D image reconstruction	No real-time image Radiative Poor spatial resolution
Intraoral Digital camera	Low cost Non-radiative	Only surface information
Periodontal probe [29]	Low cost Broad measurement range	Low sensitivity No image Invasive
OCT	High spatial resolution Real-time image 3-D image reconstruction is available	Limited penetration depth and scanning range
Raman spectroscopy [41–43]	High sensitivity Responses to mineral and chemical concentrations	*In vitro* measurement Expensive No image
Laser fluorescence spectrometer [39,44]	Real time detection Responses to bacteria and chemical concentrations	Lack of diagnostic consistency No image
